# Early life stress delays hippocampal development and diminishes the adult stem cell pool in mice

**DOI:** 10.1038/s41598-019-40868-0

**Published:** 2019-03-11

**Authors:** Mary Youssef, Piray Atsak, Jovani Cardenas, Stylianos Kosmidis, E. David Leonardo, Alex Dranovsky

**Affiliations:** 1 Department of Psychiatry, Columbia University, New York, NY 10032 USA; 2 Division of Systems Neuroscience, New York State Psychiatric Institute, New York, NY 10032 USA; 3 Graduate Program in Neurobiology and Behavior, Columbia University, New York, NY 10032 USA; 4 Department of Cognitive Neuroscience, Radboud University Medical Center, 6500 HB Nijmegen, The Netherlands; 5 Donders Institute for Brain, Cognition and Behaviour, Radboud University, 6525 EN Nijmegen, The Netherlands; 6 Department of Neuroscience, Columbia University, New York, NY 10032 USA; 7 Howard Hughes Medical Institute, Columbia University, New York, NY 10032 USA; 8 New York State Psychiatric Institute, New York, NY 10032 USA; 9 Zuckerman Mind Brain Behavior Institute, Columbia University, New York, NY 10032 USA

## Abstract

Early life stress predisposes to mental illness and behavioral dysfunction in adulthood, but the mechanisms underlying these persistent effects are poorly understood. Stress throughout life impairs the structure and function of the hippocampus, a brain system undergoing considerable development in early life. The long-term behavioral consequences of early life stress may therefore be due in part to interference with hippocampal development, in particular with assembly of the dentate gyrus (DG) region of the hippocampus. We investigated how early life stress produces long-term alterations in DG structure by examining DG assembly and the generation of a stable adult stem cell pool in routine housing and after stress induced by the limited bedding/nesting paradigm in mice. We found that early life stress leads to a more immature, proliferative DG than would be expected for the animal’s age immediately after stress exposure, suggesting that early life stress delays DG development. Adult animals exposed to early life stress exhibited a reduction in the number of DG stem cells, but unchanged neurogenesis suggesting a depletion of the stem cell pool with compensation in the birth and survival of adult-born neurons. These results suggest a developmental mechanism by which early life stress can induce long-term changes in hippocampal function by interfering with DG assembly and ultimately diminishing the adult stem cell pool.

## Introduction

Stress during early life has been consistently associated with mental illness in adulthood^1–3^, though the mechanisms underlying the persistent effects are poorly understood. In humans and in rodent experimental systems, early life stress (ELS) exposure can have detrimental consequences on adulthood hippocampal functioning, for instance dysregulation of stress reactivity, impairments in spatial learning and memory, and increases in anxiety behavior^2,4–7^. Indeed, the rodent hippocampus undergoes anatomic and cellular changes in response to stress exposure^8–10^ and hippocampal volume is reduced in humans who have experienced ELS^11,12^. Stress decreases adult hippocampal neurogenesis, which occurs in the dentate gyrus (DG)^13^. Interestingly, decreases in adult neurogenesis correlate with poorer functioning in hippocampal-dependent memory tasks^5,14^, suggesting that deficits in neurogenesis may underlie the ELS-induced cognitive impairments.

Chronic stress can have detrimental effects on hippocampal neurogenesis and functioning regardless of when during the animal’s lifetime it occurs, but ELS is more likely to induce persistent impairments compared to stress during adulthood^5,15–22^. ELS in rodent models is often administered during the first two postnatal weeks, when the DG is forming. During this time, the majority of neurons comprising the structure are born, granule cells consolidate into a well-defined layer, and stem cells become restricted to the subgranular zone (SGZ)^23–27^. Stem cell numbers and neurogenesis then decline exponentially during the subsequent year^28,29^. Since ELS coincides with the most active stages of DG development, it is intriguing to speculate that it leads to life-long dysfunction by disrupting DG formation. Previous work has shown that ELS leads to changes in DG cell proliferation and neurogenesis^10,30^, including recent reports that these measures increase shortly after ELS exposure^5,15^. These new findings are surprising because chronic stress in adulthood consistently results in decreased cell proliferation and neurogenesis^21,22,31–33^. However, the appearance of a more proliferative state in the DG after ELS could reflect developmental immaturity, which could then progress to life-long dysfunction.

In fact, both early life stress and chronic adulthood stress alter DG cell proliferation during the stress exposure^5,15,21,22,31,32,34–37^. However, interfering with stem cell division during the early postnatal period, but not later in life, leads to depletion of adult stem cells^38,39^, suggesting that the early postnatal period is critical for generating the adult stem cell pool. One intriguing and remarkably simple possibility for how ELS produces life-long DG dysfunction is that it interferes with stem cell division and DG assembly during their most active periods. However, while the effects of ELS on adult neurogenesis have been explored, the effects on stem cells have received almost no attention in the ELS literature.

In this study, we utilized the limited bedding and nesting paradigm^40^ to induce ELS from postnatal day (P)3–P10 in mouse pups expressing a short-lived Nestin reporter^41^. We found that ELS delays DG development and diminishes the adult stem cell pool in male and female mice.

## Results

### Development of the dentate gyrus from the first to second postnatal week

To understand how ELS affects DG development, we first characterized DG anatomy at the end of the first (P7) and second postnatal (P14) weeks by assessing DG volume, stem cell proliferation, and distribution of progenitor and proliferating cells. DG neural stem cells (NSCs) are radial glial-like cells that express glial fibrillary acidic protein (GFAP) and Nestin^13,23,42,43^. They divide giving rise to Nestin-expressing non-radial intermediate progenitor cells, which differentiate into neuroblasts. Neurons that develop from this lineage reside in the granule cell layer (GCL) of the DG. The stem and progenitor cells are initially spread throughout the area that will become the GCL, but then coalesce into the SGZ, where they remain into adulthood^23,24^. We utilized mice expressing a rapidly degrading Kusabira Orange fluorescent protein under the control of a Nestin promoter (Nestin-KOr)^41^ to identify stem and progenitor cells by visualization of the complete cell body and process. Identifying cell bodies of Nestin- or GFAP- expressing cells is difficult in non-transgenic mice because the endogenous proteins are restricted to the cellular processes. In these experiments, KOr+ cells include Nestin-expressing radial NSCs and non-radial intermediate progenitor cells^44,45^. Cells that express cell division marker MCM2 can be proliferating NSCs, intermediate progenitors, or neuroblasts^44,46^, as well as dividing cells unrelated to the NSC lineage.

**Figure 1:**
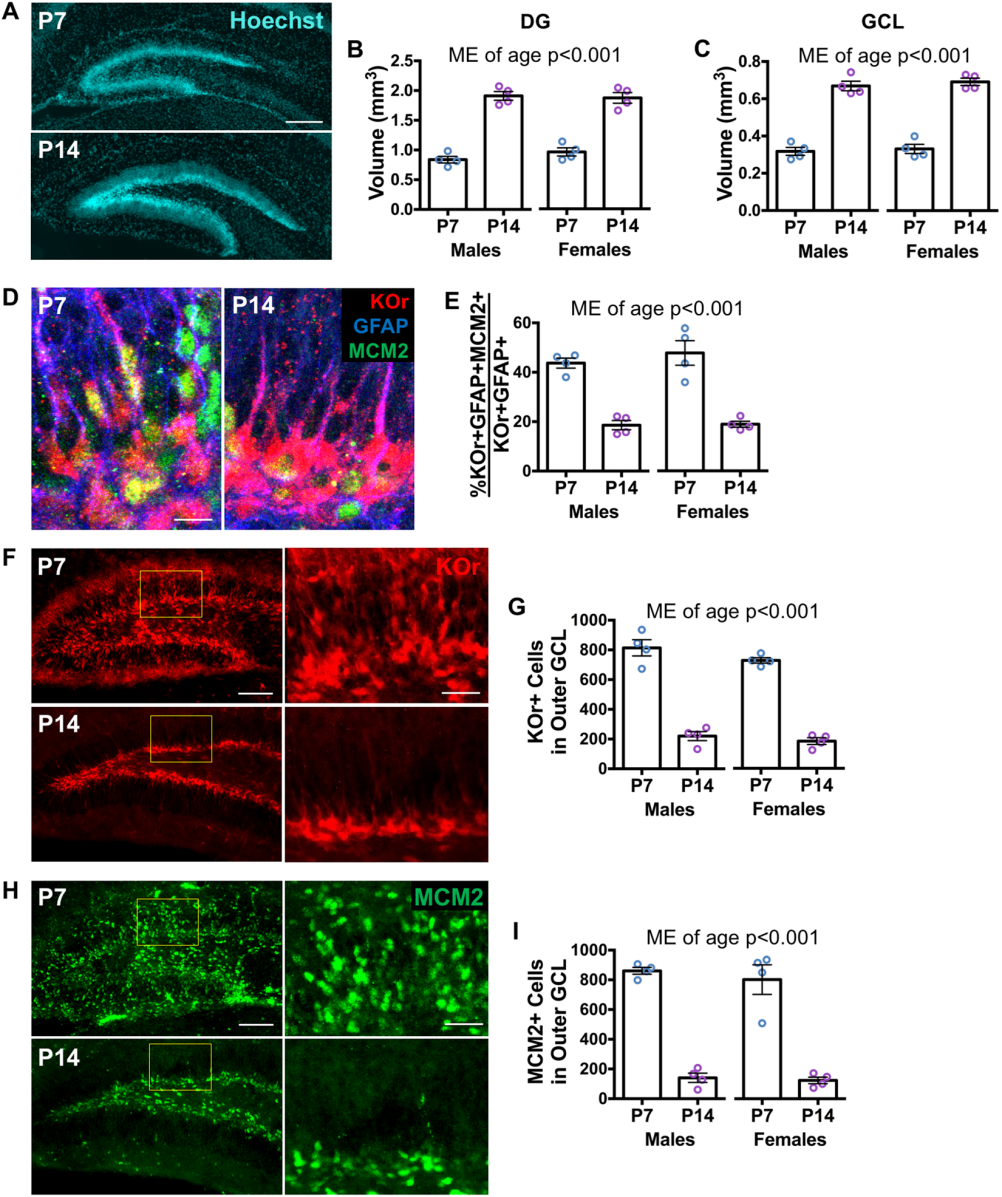
Dentate gyrus grows and consolidates during the first two postnatal weeks. (**A**) Representative images of Hoechst staining of the dentate gyrus (DG) at P7 and P14 (scale bar: 200 μm). (**B**) The volume of the DG is greater at P14 compared to at P7 (age: F_(1,12)_ = 186.9, p < 0.0001; sex: F_(1,12)_ = 0.2653, p = 0.6158; age x sex: F_(1,12)_ = 1.296, p = 0.2771). (**C**) The volume of the granule cell layer (GCL) is also greater at P14 compared to at P7 (age: F_(1,12)_ = 239.3, p < 0.0001; sex: F_(1,12)_ = 0.7126, p = 0.4151; age x sex: F_(1,12)_ = 0.0480, p = 0.8303). (**D**) Representative images of KOr+ GFAP + MCM2+ dividing neural stem cells at P7 and P14 (scale bar: 10 μm). (**E**) A smaller percentage of KOr+ GFAP+ radial stem cells express cell division marker MCM2 at P14 compared to at P7 (age: F_(1,12)_ = 86.91, p < 0.0001; sex: F_(1,12)_ = 0.5017, p = 0.4923; age x sex: F_(1,12)_ = 0.3985, p = 0.5397). (**F**) Representative images of KOr+ stem and progenitor cells including high magnification images of outlined areas at P7 and P14 (scale bars: 100 μm for low magnification and 30 μm for high magnification). (**G**) There are fewer KOr+ stem and progenitor cells in the outer third of the GCL at P14 compared to at P7 (age: F_(1,12)_ = 272.6, p < 0.0001; sex: F_(1,12)_ = 3.076, p = 0.1049; age x sex: F_(1,12)_ = 0.5044, p = 0.4911). (**H**) Representative images of MCM2+ dividing cells including high magnification images of outlined areas at P7 and P14 (scale bars: 100 μm for magnification and 30 μm for high magnification). (**I**) There are fewer MCM2 + dividing cells in the outer third of the GCL at P14 compared to at P7 (age: F_(1,12)_ = 164.5, p < 0.0001; sex: F_(1,12)_ = 0.4760, p = 0.5034; age x sex: F_(1,12)_ = 0.1396, p = 0.7152). Data are expressed as mean ± SEM. Main effect (ME) of age is noted when statistically significant (p < 0.05).

In normally developing animals, the volumes of the DG (Fig. 1A,B) and the GCL (Fig. 1A,C) approximately doubled from P7 to P14. Concurrently, the percentage of dividing stem cells, as assessed by co-localization of cell division marker MCM2 with GFAP and KOr, decreased from P7 to P14 (Fig. 1D,E). Additionally, fewer KOr+ stem and progenitor cells (Fig. 1F,G) and MCM2+ (Fig. 1H,I) dividing cells were found in the outer third of the granule cell layer at P14 versus at P7, indicating coalescence of the germinal region. No differences in the effect of age were observed between sexes or between the supra- and infrapyramidal blades of the DG (Fig. S1A–F). These results establish quantifiable measures that can be used to determine the developmental state of the DG after early life interventions. We used these measures to determine how ELS affects DG development.

### Effect of early life stress on dentate gyrus development

We exposed Nestin-KOr dams and pups to the limited bedding/nesting paradigm^40^ from P3 to P10 (Fig. S2A). This paradigm is known to result in erratic maternal care and ELS to the pups. As reported previously^5,40^, pup weight on P10 was lower in ELS animals compared to control animals (Fig. S2B). Limited bedding/nesting also led to more dam exits from the nest (Fig. S2C) and increased the amount of time pups were found outside the nest (Fig. S2D). However, the total amount of time dams engaged in nursing behavior did not differ between control and ELS cages (Fig. S2E). These results indicate that limited bedding/nesting leads to reduced quality of maternal care, but does not change the total amount of maternal care expressed by the dams.

**Figure 2:**
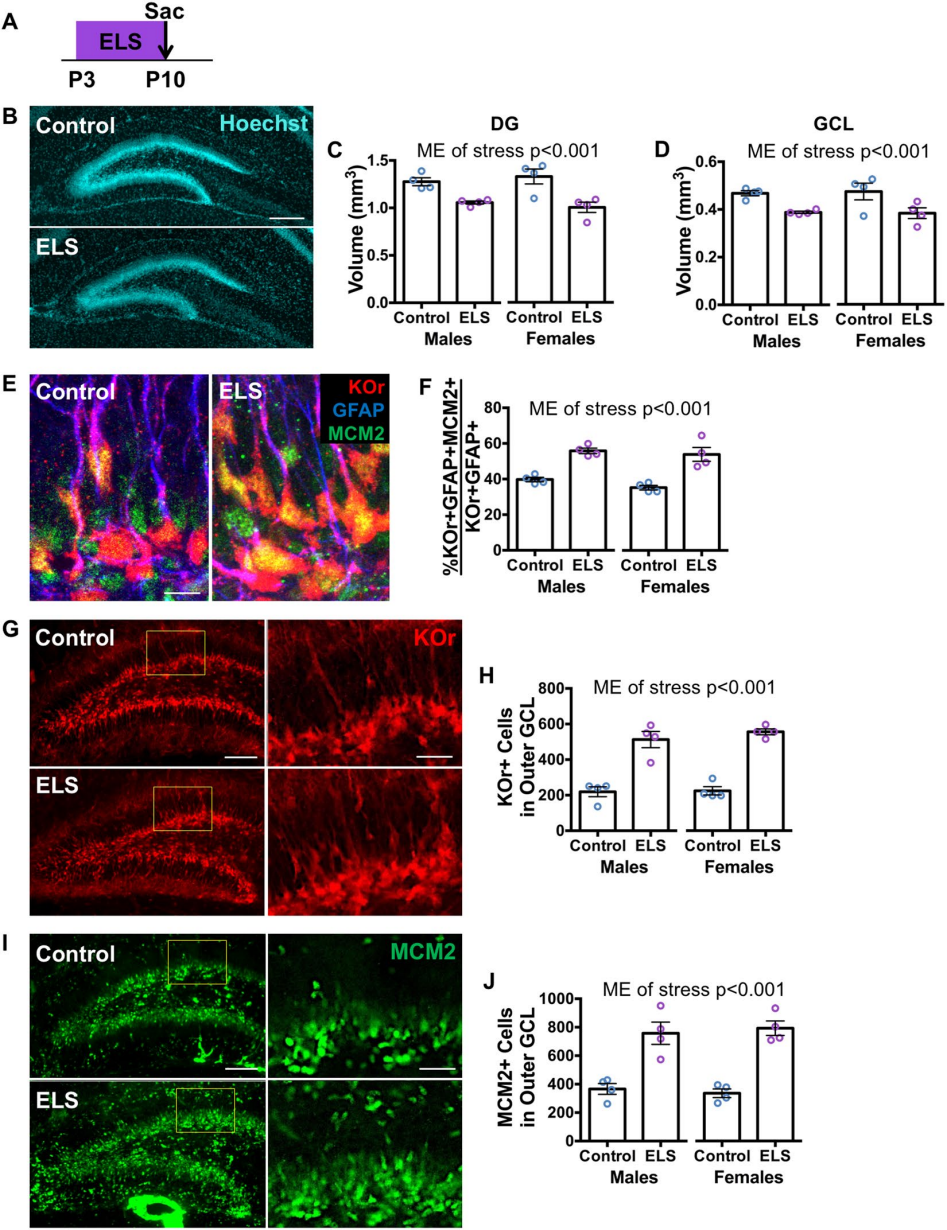
Early life stress results in an immature dentate gyrus immediately after stress exposure. (**A**) Experimental timeline denoting early life stress (ELS) from P3–P10, followed by sacrifice on P10 of Nestin-KOr animals. (**B**) Representative images of Hoechst staining of the DG in unstressed control and ELS mice (scale bar: 200 μm). (**C**) The volume of the DG is smaller in ELS animals compared to unstressed controls (stress: F_(1,12)_ = 26.56, p = 0.0002; sex: F_(1,12)_ = 0.0005, p = 0.9823; stress x sex: F_(1,12)_ = 1.017, p = 0.3332). (**D**) The volume of the GCL is also smaller in ELS animals compared to unstressed controls (stress: F_(1,12)_ = 15.74, p = 0.0019; sex: F_(1,12)_ = 0.00006, p = 0.9937; stress x sex: F_(1,12)_ = 0.0645, p = 0.8038). (**E**) Representative images of KOr + GFAP + MCM2+ dividing stem cells in unstressed control and ELS mice (scale bar: 10 μm). (**F**) A larger percentage of KOr + GFAP+ radial stem cells express cell division marker MCM2 in ELS animals compared to unstressed controls (stress: F_(1,12)_ = 58.83, p < 0.0001; sex: F_(1,12)_ = 1.856, p = 0.1981; stress x sex: F_(1,12)_ = 0.3296, p = 0.576). (**G**) Representative images of KOr+ stem and progenitor cells in unstressed control and ELS mice including high magnification images of outlined areas (scale bars: 100 μm for low magnification and 30 μm for high magnification). (**H**) There are more KOr+ stem and progenitor cells in the outer third of the GCL in ELS animals compared to unstressed controls (stress: F_(1,12)_ = 106.2, p < 0.0001; sex: F_(1,12)_ = 0.5819, p = 0.4603; stress x sex: F_(1,12)_ = 0.3877, p = 0.5452). Representative images of MCM2+ dividing cells in unstressed control and ELS mice including high magnification images of outlined areas (scale bars: 100 μm for low magnification and 30 μm for high magnification). (**J**) There are more MCM2+ dividing cells in the outer third of the GCL in ELS animals compared to unstressed controls (stress: F_(1,12)_ = 64.99, p < 0.0001; sex: F_(1,12)_ = 0.0003, p = 0.9870; stress x sex: F_(1,12)_ = 0.3791, p = 0.5496). Data are expressed as mean ± SEM. ME of stress is noted when statistically significant (p < 0.05).

We then analyzed the DG of mice that underwent ELS from P3 to P10 and were sacrificed on P10 using the same measures used to assess normal development (Fig. 2A). ELS mice had lower DG (Fig. 2B,C) and GCL (Fig. 2B,D) volumes compared to same age unstressed control mice. The percentage of dividing stem cells was greater in ELS mice compared to unstressed controls (Fig. 2E,F). ELS mice also had more KOr+ stem and progenitor cells (Fig. 2G,H) and MCM2+ dividing cells (Fig. 2I,J) in the outer third of the GCL than did control mice. Again, no main effect of sex or DG blade were detected for any of these measures (Fig. S3A–G). Together, the results indicate that ELS mice have a more immature DG after stress exposure, suggesting that ELS delays early DG development.

### Effect of early life stress on adult DG stem cells and neurogenesis

**Figure 3:**
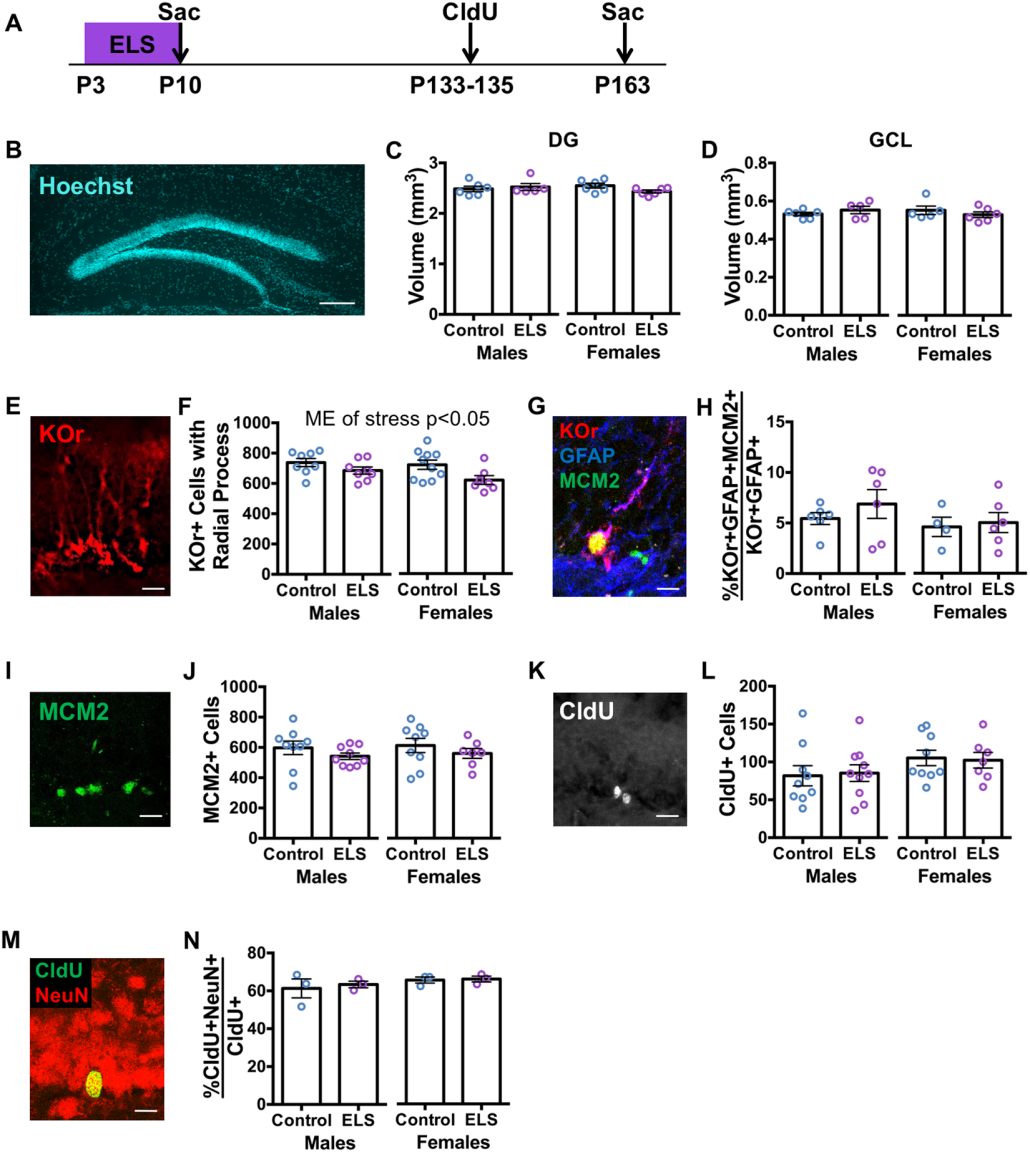
Early life stress leads to a diminished stem cell pool without altering cell proliferation and neurogenesis in young adult mice. (**A**) Experimental timeline of ELS from P3–P10, followed by chlorodeoxyuridine (CldU) administration from P133 to P135 and sacrifice on P163 of Nestin-KOr animals. (**B**) Representative image of Hoechst staining of the DG (scale bar: 200 μm). (**C**) No difference is detected in the volume of the DG between unstressed control and ELS animals at P163 (stress: F_(1,19)_ = 0.6018, p = 0.4474; sex: F_(1,19)_ = 0.3521, p = 0.5599; stress x sex: F_(1,19)_ = 2.258, p = 0.1494). (**D**) No difference is detected in the volume of the GCL between unstressed control and ELS animals (stress: F_(1,18)_ = 0.0034, p = 0.9545; sex: F_(1,18)_ = 0.0074, p = 0.9326; stress x sex: F_(1,18)_ = 1.769, p = 0.2001). (**E**) Representative image of KOr+ radial stem cells (scale bar: 20 μm). (**F**) There are fewer KOr+ radial stem cells in the DG of ELS animals compared to unstressed controls at P163 (stress: F_(1,29)_ = 7.351, p = 0.0111; sex: F_(1,29)_ = 2.391, p = 0.1329; stress x sex: F_(1,29)_ = 0.7132, p = 0.4053). (**G**) Representative image of KOr + GFAP + MCM2+ radial stem cell (scale bar: 10 μm). (**H**) No difference is detected in the percentage of KOr + GFAP+ radial stem cells expressing cell division marker MCM2 between unstressed control and ELS animals (stress: F_(1,18)_ = 0.7519, p = 0.3973; sex: F_(1,18)_ = 1.509, p = 0.2350; stress x sex: F_(1,18)_ = 0.2200, p = 0.6447). (**I**) Representative image of MCM2+ dividing cells (scale bar: 20 μm). (**J**) No difference is detected in the number of MCM2+ dividing cells in the DG between unstressed control and ELS animals (stress: F_(1,30)_ = 1.975, p = 0.1701; sex: F_(1,30)_ = 0.1582, p = 0.6937; stress x sex: F_(1,30)_ = 0.0017, p = 0.9679). (**K**) Representative image of CldU+ newborn cells (scale bar: 20 μm). (**L**) No difference is detected in the number of CldU+ newborn cells in the DG between unstressed control and ELS animals (stress: F_(1,31)_ = 0.0007, p = 0.9797; sex: F_(1,31)_ = 3.085, p = 0.0889; stress x sex: F_(1,31)_ = 0.0785, p = 0.7813). (**M**) Representative image of CldU + NeuN+ mature neuron (scale bar: 10 μm). (**N**) No difference is detected in the percentage of CldU+ cells expressing mature neuron marker NeuN between unstressed control and ELS animals (stress: F_(1,8)_ = 0.2103, p = 0.6587; sex: F_(1,8)_ = 1.440, p = 0.2644; stress × sex: F_(1,8)_ = 0.0775, p = 0.7877). Data are expressed as mean ± SEM. ME of stress is noted when statistically significant (p < 0.05).

Since our data demonstrated that ELS alters development of the DG, we hypothesized that ELS may lead to long-term effects on the adult DG. We exposed Nestin-KOr animals to ELS and assessed their DG structure on P163 (Fig. 3A,B). No differences were detected in the volume of the DG (Fig. 3C) or GCL (Fig. 3D) between control and ELS mice, indicating that the adult structure is not grossly affected by ELS.

Cellular analyses revealed that ELS mice had fewer KOr+ radial stem cells compared to controls (Fig. 3E,F) in both the suprapyramidal (Fig. S4B) and infrapyramidal (Fig. S4C) blades of the DG. No differences were detected in the percentage of KOr + GFAP+ radial stem cells that were dividing in ELS versus control mice (Fig. 3G,H) in either the suprapyramidal (Fig. S4D) or infrapyramidal (Fig. S4E) blades. No main effect of sex was detected for either of these measures. These data indicate that ELS results in a diminished adult stem cell pool.

We then quantified the number of proliferating cells and surviving newborn neurons in the adult DG to assess the effect of ELS on adult neurogenesis. No difference was detected in the total number of MCM2+ dividing cells between control and ELS mice (Fig. 3I,J). We injected CldU to label dividing cells and assessed four-week survival of newborn neurons (Fig. 3A). No difference was detected in the number of CldU+ cells in control versus ELS mice (Fig. 3K,L). Further, no difference was detected in the percentage of CldU+ cells co-expressing the mature neuron marker, NeuN, between control and ELS mice (Fig. 3M,N). No main effect of sex or DG blade (Fig. S4F-K) were observed for any of these measures. Together, the data demonstrate that while ELS leads to a reduced size of the adult stem cell pool, it does not alter cell proliferation or neuronal survival at this age.

## Discussion

We describe fundamental aspects of normal DG maturation and demonstrate that DG development is delayed by ELS by comparing the effects of ELS to the trends of normal development. Previous work using models of ELS in mice and rats reported that chronic ELS leads to higher numbers of proliferating cells and neuroblasts shortly after the stressor^5,15,47^. These findings initially seemed at odds with the established literature that stress exposure reduces neurogenesis^33–37^. However, in these previously published studies, cell proliferation was measured at an age when it is normally waning^25,28^. Our results suggest that increased proliferation in a smaller DG likely reflects an earlier developmental stage when more robust neurogenesis is necessary to complete DG growth. Our more holistic assessment of DG development revealed that ELS delays DG maturation rather than increases neurogenesis as previously suggested.

While the consequences of ELS on the DG at the end of stress exposure are robust, the effects are more modest in the adult animals, suggesting at least partial recovery of the developmental phenotype. Similar to others who looked early in adulthood^5,15,47,48^, we did not detect differences in neurogenesis in 5-month-old animals that were exposed to ELS. Nevertheless, the adult NSC pool was diminished in our 5-month-old animals. Of note, studies report that ELS can impact neurogenesis in older animals and that the effects are more notable in males^5,15^. Perhaps the decline in NSCs that we see in our adult animals contributes to the ELS-induced decline in neurogenesis later in life. Moreover, in our study, the effect of ELS on stem cell number appears to be driven by the female animals, although there was no statistical effect of sex. Together, the results suggest that both age and sex may play a role in determining the lasting effects of ELS on the hippocampal stem cell system and its lineages.

Chronic stress during adulthood is known to alter stem cell number and activity^49,50^, but our work is the first to demonstrate an effect of ELS on adult DG stem cells. We show that ELS leads to fewer adult NSCs with unchanged rates of proliferation, suggesting that NSCs produce fewer direct progeny in ELS animals. NSC progeny include intermediate progenitors that subsequently divide to give rise to neuroblasts, which continue to divide and differentiate into immature neurons^44–46^. Given that there are likely fewer intermediate progenitors being born, but that the total number of dividing cells is unchanged, there must be increased proliferation and/or survival within the pool of non-NSC dividing cells, specifically intermediate progenitors and neuroblasts. Thus, our results suggest that, in the adult DG of ELS animals, compensation occurs at the level of intermediate progenitors or neuroblasts to allow for a diminished stem cell pool to generate a comparable number of surviving neurons. Others have found that neurogenesis is decreased in 8- to 15-month animals after ELS^5,15,51^, suggesting that the capacity for compensation wanes as the animal ages. In fact, the ability of the DG niche to support neurogenesis normally diminishes in the aging animal^52,53^ and molecular interventions can accelerate this process^54^. Perhaps, this waning support is what reveals the effects of ELS on neurogenesis later in life.

Our findings of immediate and sustained structural effects of ELS exposure on the DG suggest a developmental mechanism by which ELS can induce long-term changes in hippocampal function. Developmental delay induced by ELS may alter adulthood hippocampal cellular composition, connectivity, and activity levels ultimately having long-term and possibly delayed consequences for behavior. Our work highlights the importance of investigating DG structure and function at several time points during life to understand how ELS leads to long-term impairments.

## Materials and Methods

### Animals

All mice were maintained on a 12:12 light cycle (6 am to 6 pm light) and given food and water *ad libitum*. All procedures were performed in the light cycle and complied with NIH Association for Assessment and Accreditation of Laboratory Animal Care (AAALAC) guidelines under a protocol approved by the NY State Psychiatric Institute IACUC.

#### Breeding

Transgenic mice expressing a PEST-tagged Kusabira Orange fluorescent protein under control of the Nestin promoter (Nestin-KOr)^41^ were used. Mice were maintained on a C57Bl/6 background. Nestin-KOr^+/+^ males were crossed to C57BL/6J females (Jackson Laboratories, Cat# 000664) to generate Nestin-KOr^+/−^ experimental animals. Genotypes were established using PCR as described previously^41^. Experimental mice for assessment of normal development were euthanized on P7 or P14. ELS control and stress experimental mice were euthanized on P10 or P163. Both male and female experimental animals were used.

#### Early Life Stress

ELS was performed as previously reported^40^, with minor modification to the paradigm’s start and end dates. Inexperienced Nestin-KOr^+/+^ male and C57Bl/6 female breeders were mated at 11 weeks of age to produce experimental mice. On the morning of P3 (with P0 being the morning pups were found), dams and litters were transferred to a new cage. Each cage housed one dam and litter. Control cages were set up as standard cages, with the floor covered with wood-chip bedding and one square piece of cotton nesting material (5 cm × 5 cm). In ELS cages, the floor was covered with a standard amount of wood-chip bedding, but a fine-gauge stainless steel mesh was placed 3 cm above the cage floor. One-half square of cotton nesting material was placed above the steel mesh. Dams had *ad libitum* access to food and water. Both groups were left completely undisturbed until P10. On the morning of P10, all pups were weighed and entire litters were either euthanized or returned to standard cages with dams for survival to adulthood. Pups were weaned on P25, with control and stress mice remaining in separate cages. Mice were separated by sex at weaning.

Maternal behavior was scored three times per day from P3–P9, twice during the light phase (8 am, 1 pm) and once during the dark phase (6:15 pm). Scoring sessions lasted one hour. Maternal behavior and presence of pups outside the nest were observed in each cage once every three minutes, for a total of 20 observations per session. Maternal behaviors scored included arched back nursing, passive nursing, licking and grooming of the pups, contact with the pups, and no contact/being outside the nest. Arched back nursing, passive nursing, and licking and grooming were considered nursing behaviors. The number of observations per scoring session during which dams exhibited nursing behavior or during which one or more pups was found outside the nest, as well as the number of dam exits from the nest, were analyzed. Maternal behavior data are reported as the number of events per one-hour scoring session averaged across the three scoring sessions per day.

#### CldU Injections

Adult ELS control and stress experimental mice were injected intraperitoneally (i.p.) with chlorodeoxyuridine (CldU, Sigma-Aldrich, Cat# C6891) at a dose of 100 mg/kg twice daily for 3 days from P133–P135. Mice were euthanized on P163 (4 weeks after the last CldU administration).

### Tissue Preparation

Mice were anesthetized with a mixture of 150 mg/kg ketamine and 10 mg/kg xylazine and perfused transcardially with ice cold phosphate buffered saline (PBS), followed by 4% paraformaldehyde (PFA) in PBS. Brains were removed and postfixed by immersion in 4% PFA for 24 hours, then switched to 30% sucrose in PBS for 48 hours for cryoprotection. Whole heads for animals euthanized at ages P7, P10, and P14 were stored in 4% PFA for an additional 24 hours before brain extraction to minimize damage during removal from skull. Brains were sectioned coronally on a cryostat to prepare 35 μm sections, which were stored in PBS with 0.02% sodium azide at 4 °C. Tissue was serially sectioned into six wells per brain.

### Immunohistochemistry

Free floating tissue sections were triple washed in PBS, then incubated for 1 hour in blocking solution (12.5% normal donkey serum and 0.25% triton in PBS), followed by incubation with one or more of the following primary antibodies diluted in blocking solution: goat anti-MCM2 1:100 (Santa Cruz Biotechnology, Cat# sc-9839), mouse anti-GFAP 1:500 (Millipore, Cat# MAB3402), rabbit anti-Kusabira Orange 1:500 (MBL International, Cat# PM051), rat anti-BrdU 1:2500 (Accurate Chemical, Cat# OBT0030G), mouse anti-NeuN 1:1000 (Millipore, Cat# MAB377). Before incubation in blocking solution, tissue being stained with anti-BrdU antibody underwent antigen retrieval by pre-treatment with 2N hydrochloric acid (HCl) at 37 °C for 30 minutes, followed by 0.1M boric acid at room temperature for 10 minutes and three PBS washes. Tissue was incubated with primary antibodies at 4 °C overnight or for 40 hours with anti-BrdU. Following primary antibody incubation, tissue was triple washed in PBS and incubated for one hour with donkey fluorescent secondary antibodies (Jackson ImmunoResearch) diluted 1:400 in PBS with Hoechst 1:10,000 (ThermoFisher Scientific, Cat# 33342). After secondary antibody incubation, tissue was triple washed in PBS, mounted onto slides and coverslipped with Aqua-Poly/Mount (VWR, Cat# 87001-902).

### Imaging and Cell Quantification

#### DG and GCL Volume

One hemisphere of every 6^th^ section across the entire anterior-posterior axis was assessed. Based on Hoechst counterstain using a 4x or 10x objective on an epifluorescence microscope (Olympus IX71), the DG (GCL, hilus, and molecular layer) or GCL were manually outlined and area was measured using Olympus cellSense Dimension Software (https://www.olympus-lifescience.com/en/software/cellsens/). The area of the DG and GCL in one hemisphere of every 6^th^ section was summed and multiplied by 6 and 0.035 (section thickness in mm) to determine the volume of one DG or GCL.

#### Stem Cell Proliferation

For P7, P10, and P14 animals, three sections equally spaced across the anterior-posterior axis of the DG were selected per animal using Hoechst counterstain with the 10x objective on the epifluorescence microscope. One 63x frame was captured using a confocal microscope (Leica TCS SP8) for each blade (suprapyramidal and infrapyramidal) of the DG of each selected section for a total of six frames. For P163 animals, four sections equally spaced across the anterior-posterior axis of the DG were selected per animal using Hoechst counterstain with the 10x objective on the epifluorescence microscope. The entire DG in one hemisphere of each selected section was imaged on the confocal microscope. Confocal images were visualized using Leica LAS X Core software (http://www.leica-microsystems.com/products/microscope-software/details/product/leica-las-x-ls/). All KOr + GFAP+ cells with radial processes were manually counted in all captured frames and MCM2 co-expression was manually assessed. For P7, P10, and P14 animals, 200 to 300 KOr + GFAP+ radial stem cells were assessed per animal (average: 250 cells/animal). For P163 animals, 150 to 300 KOr + GFAP+ radial stem cells were assessed per animal (average: 220 cells/animal).

#### KOr+ and MCM2+ Cells in the Outer GCL

Five sections equally spaced across the anterior-posterior axis of the DG were selected per animal by visualizing Hoechst counterstain using a 10x objective on the epifluorescence microscope. Based on Hoechst counterstain using the 20x objective on the epifluorescence microscope, the outer third of the GCL in one frame was manually outlined during live visualization using Olympus cellSense Dimension Software. The number of MCM2+ cells and KOr+ cells in the outlined area were manually counted using the 20x objective during live visualization using cellSense Dimension software.

#### KOr+, MCM2+, and CldU+ Cells in the Adult DG

Every 6^th^ section across the entire anterior-posterior axis was assessed. For KOr and MCM2 cell counts, one hemisphere per section was assessed. For CldU counts, both hemispheres in each section were assessed. All KOr+ cells with somas in the SGZ and radial processes were manually counted using the 40x objective during live visualization on the epifluorescence microscope. All MCM2+ cells in the SGZ were manually counted using the 40x objective during live visualization on the epifluorescence microscope. All CldU+ cells in the SGZ and GCL were manually counted using the 20x objective during live visualization on the epifluorescence microscope.

#### Neuronal Phenotyping of CldU+ Cells in the Adult DG

Both hemispheres of every 6^th^ section across the entire anterior-posterior axis was assessed. All CldU+ cells in the SGZ and GCL was manually identified during live visualization on a Leica TCS SP8 episcope using the 40x objective. CldU+ cells were then visualized with confocal imaging using LAS X software (https://www.leica-microsystems.com/products/confocal-microscopes/details/product/leica-tcs-sp8/). Co-expression of NeuN was manually assessed during live confocal imaging using the 40x objective. Sixty to 230 CldU+ cells were assessed per animal (average: 135 cells/animal).

### Statistical Analysis

Data were analyzed using GraphPad Prism Software (https://www.graphpad.com/scientific-software/prism/). All data sets are presented as mean ± standard error of the mean (SEM). Statistical significance was set at p < 0.05.

Immunofluorescence and animal weight data were analyzed using two-way ANOVA with two fixed factors: condition (sacrifice age: P7 vs. P14; or stress: control vs. ELS) and sex (male vs. female). Maternal behavior data were analyzed using two-way repeated measures ANOVA with two fixed factors: stress (control vs. ELS) and day (P3–P9).

## Supplementary information

Supplementary Figures

## Data Availability

The datasets generated during and analyzed during the current study are available from the corresponding author on reasonable request.

## References

[1] Heim C, Nemeroff CB (2001). The role of childhood trauma in the neurobiology of mood and anxiety disorders: preclinical and clinical studies. Biol Psychiatry.

[2] de Kloet ER, Sibug RM, Helmerhorst FM, Schmidt MV (2005). Stress, genes and the mechanism of programming the brain for later life. Neurosci Biobehav Rev.

[3] Green JG (2010). Childhood adversities and adult psychiatric disorders in the national comorbidity survey replication I: associations with first onset of DSM-IV disorders. Arch Gen Psychiatry.

[4] Huot RL, Plotsky PM, Lenox RH, McNamara RK (2002). Neonatal maternal separation reduces hippocampal mossy fiber density in adult Long Evans rats. Brain Res.

[5] Naninck EF (2015). Chronic early life stress alters developmental and adult neurogenesis and impairs cognitive function in mice. Hippocampus.

[6] Sapolsky RM, Meaney MJ (1986). Maturation of the adrenocortical stress response: neuroendocrine control mechanisms and the stress hyporesponsive period. Brain Res.

[7] Wilkinson PO, Goodyer IM (2011). Childhood adversity and allostatic overload of the hypothalamic-pituitary-adrenal axis: a vulnerability model for depressive disorders. Dev Psychopathol.

[8] Gould E, Woolley CS, McEwen BS (1991). Adrenal steroids regulate postnatal development of the rat dentate gyrus: I. Effects of glucocorticoids on cell death. J Comp Neurol.

[9] Gould E, Woolley CS, Cameron HA, Daniels DC, McEwen BS (1991). Adrenal steroids regulate postnatal development of the rat dentate gyrus: II. Effects of glucocorticoids and mineralocorticoids on cell birth. J Comp Neurol.

[10] Lajud N, Torner L (2015). Early life stress and hippocampal neurogenesis in the neonate: sexual dimorphism, long term consequences and possible mediators. Front Mol Neurosci.

[11] Vythilingam M (2002). Childhood trauma associated with smaller hippocampal volume in women with major depression. Am J Psychiatry.

[12] Bremner JD (1997). Magnetic resonance imaging-based measurement of hippocampal volume in posttraumatic stress disorder related to childhood physical and sexual abuse–a preliminary report. Biological psychiatry.

[13] Ming GL, Song H (2005). Adult neurogenesis in the mammalian central nervous system. Annu Rev Neurosci.

[14] Marin-Burgin A, Schinder AF (2012). Requirement of adult-born neurons for hippocampus-dependent learning. Behav Brain Res.

[15] Suri D (2013). Early stress evokes age-dependent biphasic changes in hippocampal neurogenesis, BDNF expression, and cognition. Biol Psychiatry.

[16] Mirescu C, Peters JD, Gould E (2004). Early life experience alters response of adult neurogenesis to stress. Nat Neurosci.

[17] Aisa B (2009). Effects of neonatal stress on markers of synaptic plasticity in the hippocampus: implications for spatial memory. Hippocampus.

[18] Kikusui T, Mori Y (2009). Behavioural and neurochemical consequences of early weaning in rodents. J Neuroendocrinol.

[19] Leslie AT (2011). Impact of early adverse experience on complexity of adult-generated neurons. Transl Psychiatry.

[20] Hulshof HJ (2011). Maternal separation decreases adult hippocampal cell proliferation and impairs cognitive performance but has little effect on stress sensitivity and anxiety in adult Wistar rats. Behav Brain Res.

[21] Elizalde N (2010). Sustained stress-induced changes in mice as a model for chronic depression. Psychopharmacology (Berl).

[22] Lagace DC (2010). Adult hippocampal neurogenesis is functionally important for stress-induced social avoidance. Proc Natl Acad Sci USA.

[23] Nicola Z, Fabel K, Kempermann G (2015). Development of the adult neurogenic niche in the hippocampus of mice. Front Neuroanat.

[24] Sugiyama T, Osumi N, Katsuyama Y (2013). The germinal matrices in the developing dentate gyrus are composed of neuronal progenitors at distinct differentiation stages. Dev Dyn.

[25] Stanfield BB, Cowan WM (1979). The development of the hippocampus and dentate gyrus in normal and reeler mice. J Comp Neurol.

[26] Muramatsu R, Ikegaya Y, Matsuki N, Koyama R (2007). Neonatally born granule cells numerically dominate adult mice dentate gyrus. Neuroscience.

[27] Navarro-Quiroga I, Hernandez-Valdes M, Lin SL, Naegele JR (2006). Postnatal cellular contributions of the hippocampus subventricular zone to the dentate gyrus, corpus callosum, fimbria, and cerebral cortex. J Comp Neurol.

[28] Ben Abdallah NM, Slomianka L, Vyssotski AL, Lipp HP (2010). Early age-related changes in adult hippocampal neurogenesis in C57 mice. Neurobiol Aging.

[29] Encinas JM (2011). Division-coupled astrocytic differentiation and age-related depletion of neural stem cells in the adult hippocampus. Cell Stem Cell.

[30] Suri D, Vaidya VA (2015). The adaptive and maladaptive continuum of stress responses - a hippocampal perspective. Rev Neurosci.

[31] Veena J (2009). Enriched environment restores hippocampal cell proliferation and ameliorates cognitive deficits in chronically stressed rats. Journal of neuroscience research.

[32] Liu Q (2008). Repeated clomipramine treatment reversed the inhibition of cell proliferation in adult hippocampus induced by chronic unpredictable stress. The pharmacogenomics journal.

[33] Heine VM, Maslam S, Zareno J, Joels M, Lucassen PJ (2004). Suppressed proliferation and apoptotic changes in the rat dentate gyrus after acute and chronic stress are reversible. The European journal of neuroscience.

[34] Oreland S, Nylander I, Pickering C (2010). Prolonged maternal separation decreases granule cell number in the dentate gyrus of 3-week-old male rats. Int J Dev Neurosci.

[35] Baek SB (2011). The phosphodiesterase type-5 inhibitor, tadalafil, improves depressive symptoms, ameliorates memory impairment, as well as suppresses apoptosis and enhances cell proliferation in the hippocampus of maternal-separated rat pups. Neurosci Lett.

[36] Lajud N, Roque A, Cajero M, Gutierrez-Ospina G, Torner L (2012). Periodic maternal separation decreases hippocampal neurogenesis without affecting basal corticosterone during the stress hyporesponsive period, but alters HPA axis and coping behavior in adulthood. Psychoneuroendocrinology.

[37] Baek SS (2012). Effects of postnatal treadmill exercise on apoptotic neuronal cell death and cell proliferation of maternal-separated rat pups. Brain Dev.

[38] Kirshenbaum GS, Lieberman SR, Briner TJ, Leonardo ED, Dranovsky A (2014). Adolescent but not adult-born neurons are critical for susceptibility to chronic social defeat. Front Behav Neurosci.

[39] Youssef M (2018). Ablation of proliferating neural stem cells during early life is sufficient to reduce adult hippocampal neurogenesis. Hippocampus.

[40] Rice CJ, Sandman CA, Lenjavi MR, Baram TZ (2008). A novel mouse model for acute and long-lasting consequences of early life stress. Endocrinology.

[41] Kanki H, Shimabukuro MK, Miyawaki A, Okano H (2010). “Color Timer” mice: visualization of neuronal differentiation with fluorescent proteins. Mol Brain.

[42] Bonaguidi MA (2011). *In vivo* clonal analysis reveals self-renewing and multipotent adult neural stem cell characteristics. Cell.

[43] Lagace DC (2007). Dynamic contribution of nestin-expressing stem cells to adult neurogenesis. J Neurosci.

[44] Von Bohlen Und Halbach O (2007). Immunohistological markers for staging neurogenesis in adult hippocampus. Cell Tissue Res.

[45] Fukuda S (2003). Two distinct subpopulations of nestin-positive cells in adult mouse dentate gyrus. J Neurosci.

[46] Hodge RD (2008). Intermediate progenitors in adult hippocampal neurogenesis: Tbr2 expression and coordinate regulation of neuronal output. J Neurosci.

[47] Nair A (2007). Stressor-specific regulation of distinct brain-derived neurotrophic factor transcripts and cyclic AMP response element-binding protein expression in the postnatal and adult rat hippocampus. Neuropsychopharmacology.

[48] Kanatsou S (2017). Overexpression of Mineralocorticoid Receptors in the Mouse Forebrain Partly Alleviates the Effects of Chronic Early Life Stress on Spatial Memory, Neurogenesis and Synaptic Function in the Dentate Gyrus. Front Cell Neurosci.

[49] Dranovsky A (2011). Experience dictates stem cell fate in the adult hippocampus. Neuron.

[50] Song J (2012). Neuronal circuitry mechanism regulating adult quiescent neural stem-cell fate decision. Nature.

[51] Ruiz R (2018). Early life stress accelerates age-induced effects on neurogenesis, depression, and metabolic risk. Psychoneuroendocrinology.

[52] Apple DM, Solano-Fonseca R, Kokovay E (2017). Neurogenesis in the aging brain. Biochem Pharmacol.

[53] Conover JC, Notti RQ (2008). The neural stem cell niche. Cell Tissue Res.

[54] Ali AA (2015). Premature aging of the hippocampal neurogenic niche in adult Bmal1-deficient mice. Aging (Albany NY).

