# Supplementary material for: Early life stress delays hippocampal development and diminishes the adult stem cell pool in mice
Source: Sci Rep. 2019 Mar 11;9:4120. doi: 10.1038/s41598-019-40868-0 (PMC6412041; doi:10.1038/s41598-019-40868-0)
Supplement: Supplementary file 1 — Supplementary Figures [file 41598_2019_40868_MOESM1_ESM.docx]

**Supplementary Information**

**Early life stress delays hippocampal development and diminishes the adult stem cell pool in mice**

Mary Youssef, Piray Atsak, Jovani Cardenas, Stylianos Kosmidis, E. David Leonardo, Alex Dranovsky

**
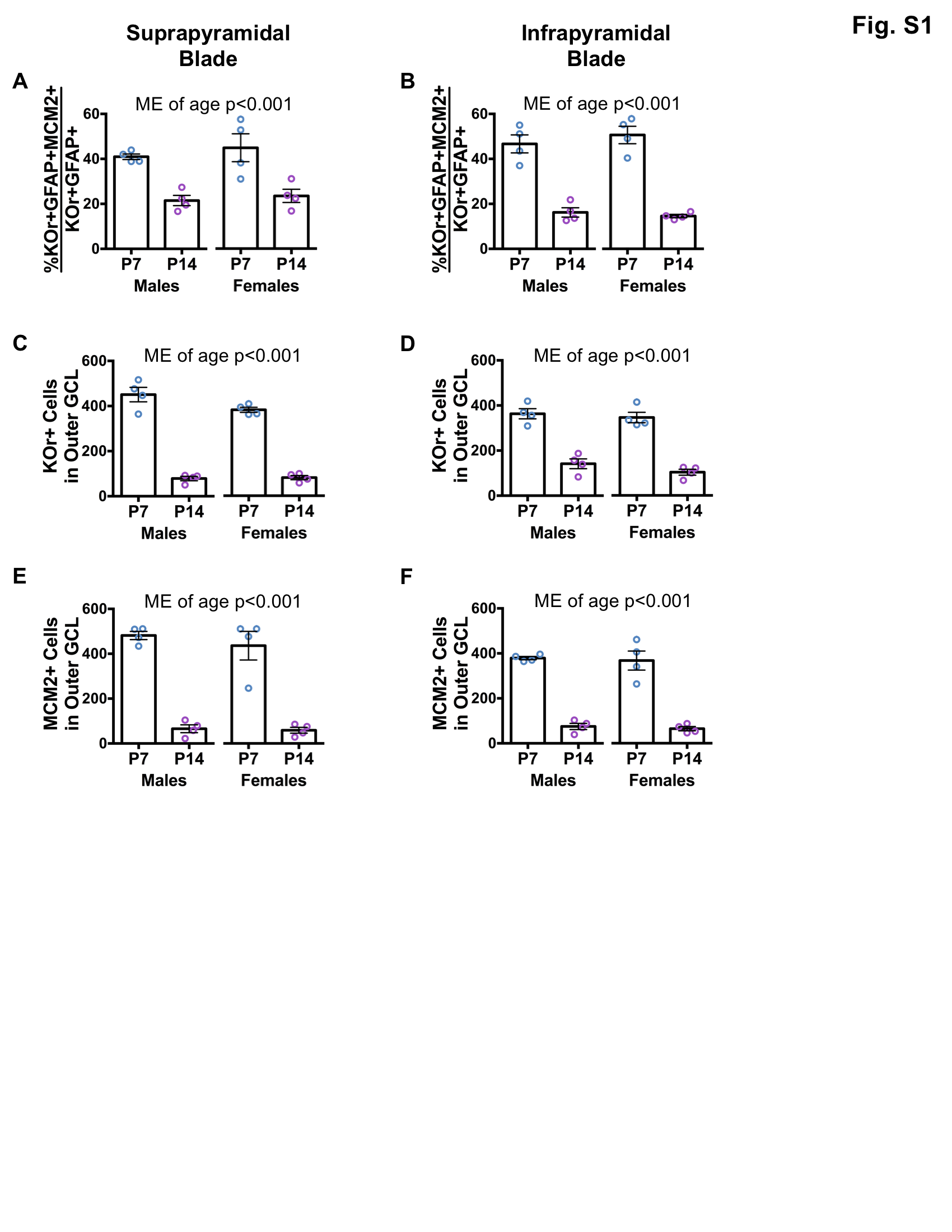
**

**Fig. S1. Development of the dentate gyrus during the first two postnatal weeks is nearly identical in the suprapyramidal and infrapyramidal blades.** A smaller percentage of KOr+GFAP+ radial stem cells express cell division marker MCM2 at P14 compared to at P7 in both the (A) suprapyramidal (age: F_(1,12)_ = 31.13, p=0.0001; sex: F_(1,12)_ = 0.5765, p=0.4624; age x sex: F_(1,12)_ = 0.0632, p=0.8058) and (B) infrapyramidal (age: F_(1,12)_ = 123.0, p<0.0001; sex: F_(1,12)_ = 0.1484, p=0.7068; age x sex: F_(1,12)_ = 0.8633, p=0.3711) blades of the DG. There are fewer KOr+ stem and progenitor cells in the outer third of both the (C) suprapyramidal (age: F_(1,12)_ = 336.2, p<0.0001; sex: F_(1,12)_ = 3.355, p=0.0919; age x sex: F_(1,12)_ = 3.766, p=0.0762) and (D) infrapyramidal (age: F_(1,12)_ = 126.9, p<0.0001; sex: F_(1,12)_ = 1.691, p=0.2179; age x sex: F_(1,12)_ = 0.2897, p=0.6002) blades of the GCL at P14 compared to at P7. There are fewer MCM2+ dividing cells in the outer third of both the (E) suprapyramidal (age: F_(1,12)_ = 129.3, p<0.0001; sex: F_(1,12)_ = 0.5784, p=0.4616; age x sex: F_(1,12)_ = 0.3132, p=0.5860) and (F) infrapyramidal (age: F_(1,12)_ = 172.2, p<0.0001; sex: F_(1,12)_ = 0.2320, p=0.6387; age x sex: F_(1,12)_ = 0.0014, p=0.9704) blades of the GCL at P14 compared to at P7. Data are expressed as mean ± SEM. ME of age is noted when statistically significant (p<0.05).

**
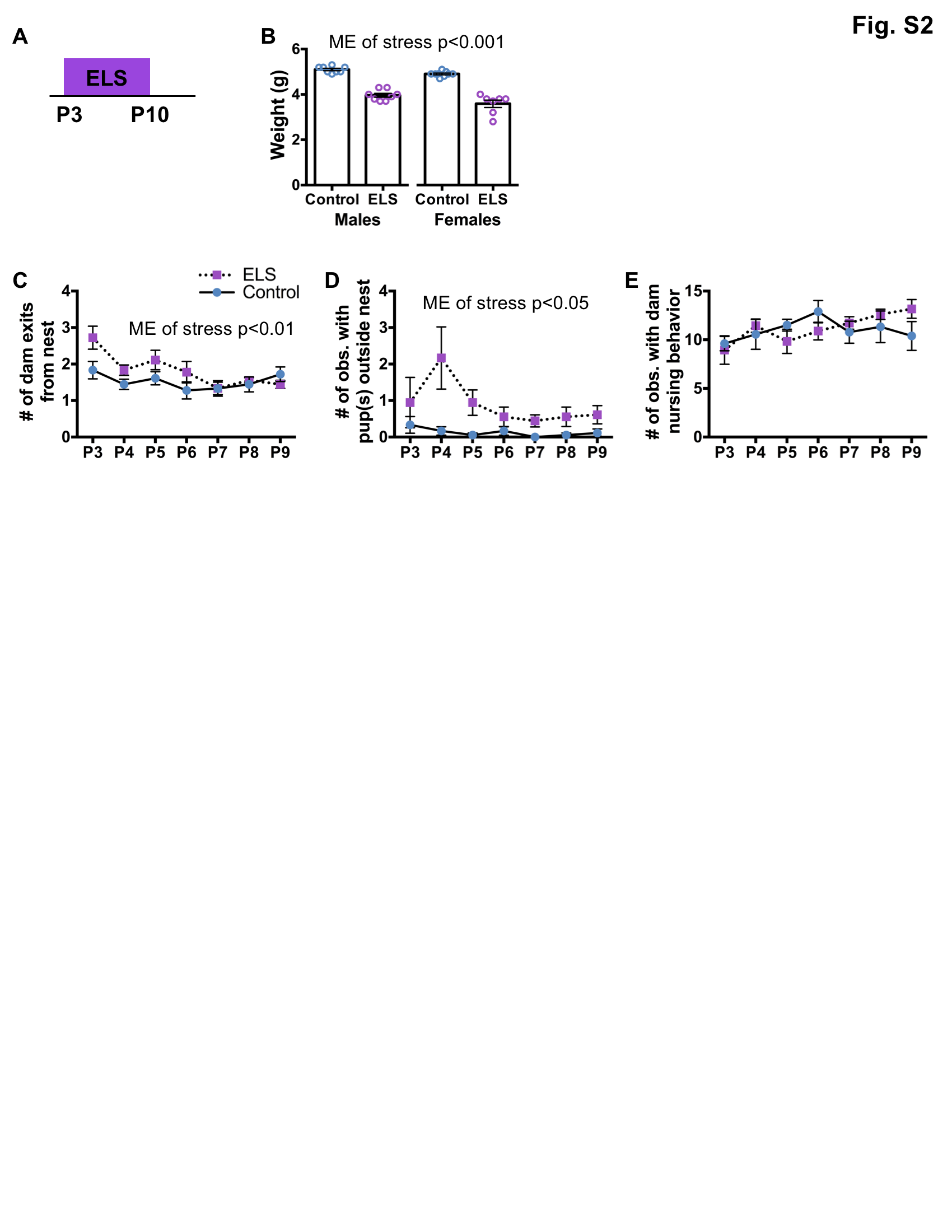
**

**Fig. S2. The** **limited bedding/nesting early life** **stress paradigm leads to decreased pup weight and erratic maternal care**. (A) Experimental timeline of ELS induced by the limited bedding/nesting paradigm from P3-P10. (B) ELS leads to decreased pup weight at P10 in ELS animals compared to unstressed controls (stress: F_(1,28)_ = 193.4, p<0.0001; sex: F_(1,28)_ = 10.70, p=0.0028; stress x sex: F_(1,28)_ = 1.005, p=0.3246). (C) Dams exit the nest more frequently in ELS cages compared to control cages (stress: F_(1,10)_ = 11.65, p=0.0066; day: F_(6,60)_ = 4.109, p=0.0016; stress x day: F_(6,60)_ = 1.593, p=0.1649). (D) ELS pups are found outside the nest more often than control pups (stress: F_(1,10)_ = 6.804, p=0.0261; day: F_(6,60)_ = 2.339, p=0.0427; stress x day: F_(6,60)_ = 1.848, p=0.1050). (E) No difference is detected in the total amount of time dams spent engaged in nursing behavior between control and ELS cages (stress: F_(1,10)_ = 0.1410, p=0.7151; day: F_(6,60)_ = 1.455, p=0.2091; stress x day: F_(6,60)_ = 1.211, p=0.3134). For maternal care data, control n=6, ELS n=6 cages. Data are expressed as mean ± SEM. ME of stress is noted when statistically significant (p<0.05).

**
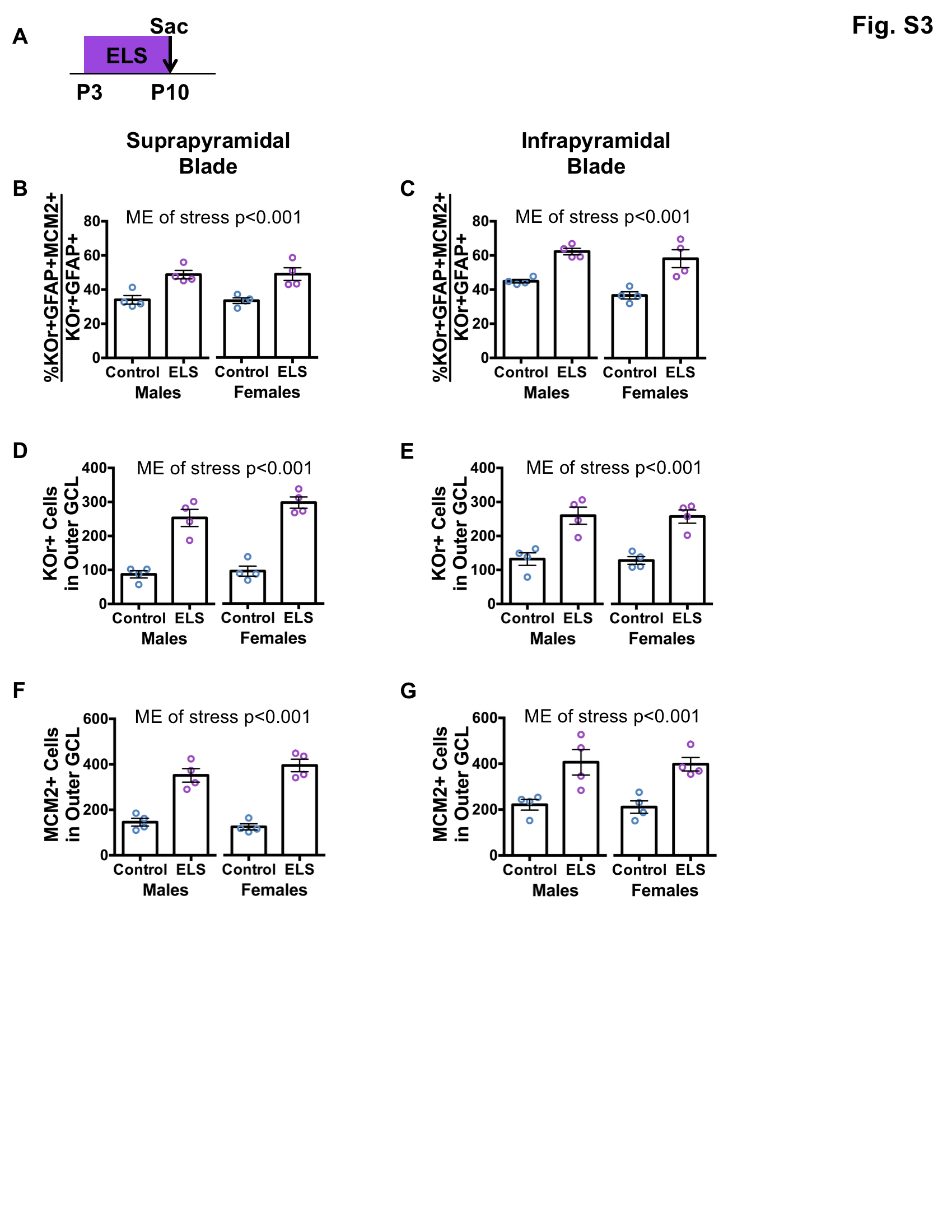
Fig. S3. Effects of early life stress on DG development occur in both the suprapyramidal and infrapyramidal blades.** (A) Experimental timeline of ELS from P3-P10, followed by sacrifice on P10 in Nestin-KOr animals. A larger percentage of KOr+GFAP+ radial stem cells express cell division marker MCM2 in ELS animals compared to unstressed controls in both the (B) suprapyramidal (stress: F_(1,12)_ = 31.79, p=0.0001; sex: F_(1,12)_ = 0.0031, p=0.9565; stress x sex: F_(1,12)_ = 0.0216, p=0.8855) and (C) infrapyramidal (stress: F_(1,12)_ = 41.37, p<0.0001; sex: F_(1,12)_ = 3.894, p= 0.0719; stress x sex: F_(1,12)_ = 0.4846, p=0.4996) blades of the DG. There are more KOr+ stem and progenitor cells in the outer third of both the (D) suprapyramidal (stress: F_(1,12)_ = 108.8, p<0.0001; sex: F_(1,12)_ = 2.281, p=0.1568; stress x sex: F_(1,12)_ = 1.057, p=0.3241) and (E) infrapyramidal (stress: F_(1,12)_ = 44.79, p<0.0001; sex: F_(1,12)_ = 0.0332, p=0.8584; stress x sex: F_(1,12)_ = 0.0015, p=0.9695) blades of the GCL in ELS animals compared to controls. There are more MCM2+ dividing cells in the outer third of both the (F) suprapyramidal (stress: F_(1,12)_ = 108.0, p<0.0001; sex: F_(1,12)_ = 0.2106, p=0.6545; stress x sex: F_(1,12)_ = 1.926, p=0.1905) and (G) infrapyramidal (stress: F_(1,12)_ = 26.54, p=0.0002; sex: F_(1,12)_ = 0.0710, p=0.7945; stress x sex: F_(1,12)_ = 0.0003, p=0.9865) blades of the GCL in ELS animals compared to controls. Data are expressed as mean ± SEM. ME of stress is noted when statistically significant (p<0.05).

**
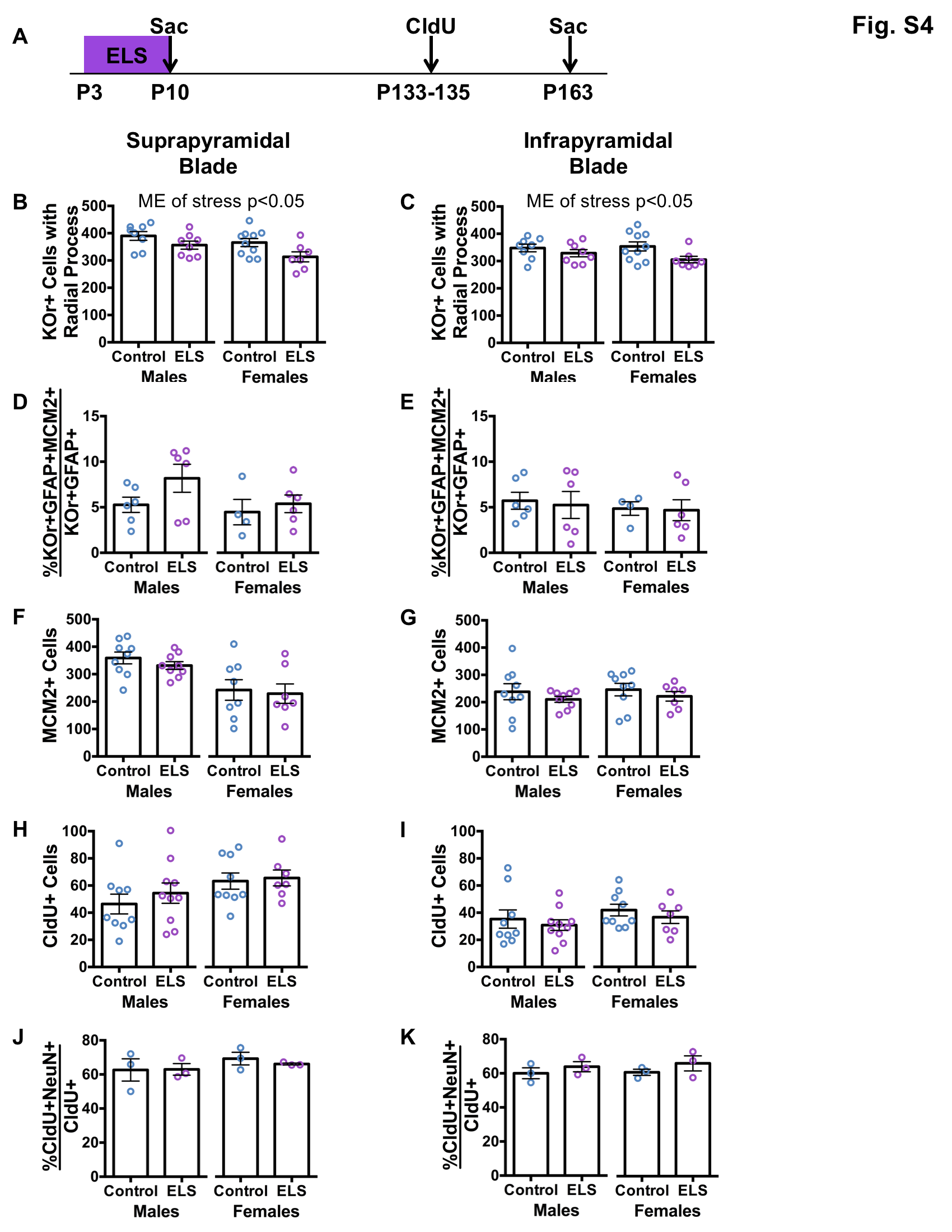
**

**Fig. S4.** **Early life stress affects adult DG stem cells and neurogenesis in the suprapyramidal and infrapyramidal blades similarly.** (A) Experimental timeline of ELS from P3-P10, followed by CldU administration from P133 to P135 and sacrifice on P163 in Nestin-KOr animals. There are fewer KOr+ radial stem cells in both the (B) suprapyramidal (stress: F_(1,29)_ 7.284, p=0.0115; sex: F_(1,29)_ = 4.360, p=0.0457; stress x sex: F_(1,29)_ = 0.3422, p=0.5631) and (C) infrapyramidal (stress: F_(1,29)_ = 5.200, p=0.0301; sex: F_(1,29)_ = 0.5003, p=0.4850; stress x sex: F_(1,29)_ = 0.9730, p=0.3321) blades of the DG of ELS animals compared to unstressed controls. No differences are detected in the percentage of KOr+GFAP+ radial stem cells expressing cell division marker MCM2 in both the (D) suprapyramidal (stress: F_(1,18)_ = 2.453, p=0.1347; sex: F_(1,18)_ = 2.188, p=0.1564; stress x sex: F_(1,18)_ = 0.6717, p=0.4232) and (E) infrapyramidal (stress: F_(1,18)_ = 0.07346, p= 0.7894; sex: F_(1,18)_ = 0.4051, p=0.5325; stress x sex: F_(1,18)_ = 0.01386, p=0.9076) blades of the DG between unstressed control and ELS animals. No differences are detected in the number of MCM2+ dividing cells in both the (F) suprapyramidal (stress: F_(1,29)_ = 0.7941, p=0.3802; sex: F_(1,29)_ = 0.0337, p=0.8556; stress x sex: F_(1,29)_ = 0.2394, p=0.6283) and (G) infrapyramidal (stress: F_(1,30)_ = 1.439, p=0.2397; sex: F_(1,30)_ = 0.1695, p=0.6835; stress x sex: F_(1,30)_ = 0.0061, p=0.9381) blades of the DG between unstressed control and ELS animals. No differences are detected in the number of CldU+ newborn cells in both the (H) suprapyramidal (stress: F_(1,31)_ = 0.5470, p=0.4651; sex: F_(1,31)_ = 4.166, p=0.0498; stress x sex: F_(1,31)_ = 0.1706, p=0.6824) and (I) infrapyramidal (stress: F_(1,31)_ = 0.9067, p=0.3484; sex: F_(1,31)_ = 1.429, p=0.2409; stress x sex: F_(1,31)_ =0.0050, p=0.9438) blades of the DG between unstressed control and ELS animals. No differences are detected in the percentage of CldU+ cells expressing mature neuron marker NeuN in both the (J) suprapyramidal (stress: F_(1,8)_ = 0.1165, p=0.7417; sex: F_(1,8)_ = 1.314, p=0.2848; stress x sex: F_(1,8)_ = 0.1774, p=0.6847) and (K) infrapyramidal (stress: F_(1,8)_ = 1.951, p=0.2001; sex: F_(1,8)_ = 0.2705, p=0.6171; stress x sex: F_(1,8)_ = 0.0491, p=0.8302) blades of the DG between unstressed control and ELS animals. Data are expressed as mean ± SEM. ME of stress is noted when statistically significant (p<0.05).
